# Artificial-Intelligence Bio-Inspired Peptide for Salivary Detection of SARS-CoV-2 in Electrochemical Biosensor Integrated with Machine Learning Algorithms

**DOI:** 10.3390/bios15020075

**Published:** 2025-01-28

**Authors:** Marcelo Augusto Garcia-Junior, Bruno Silva Andrade, Ana Paula Lima, Iara Pereira Soares, Ana Flávia Oliveira Notário, Sttephany Silva Bernardino, Marco Fidel Guevara-Vega, Ghabriel Honório-Silva, Rodrigo Alejandro Abarza Munoz, Ana Carolina Gomes Jardim, Mário Machado Martins, Luiz Ricardo Goulart, Thulio Marquez Cunha, Murillo Guimarães Carneiro, Robinson Sabino-Silva

**Affiliations:** 1 Department of Physiology, Laboratory of Nanobiotechnology—Dr. Luiz Ricardo Goulart, Innovation Center in Salivary Diagnostic and Nanobiotechnology, Institute of Biomedical Sciences, Federal University of Uberlandia (UFU), Uberlândia 38408-100, Brazil; marceloagjr@ufu.br (M.A.G.-J.); anapaulalim@ufu.br (A.P.L.); sttephanysb@ufu.br (S.S.B.); marco.guevara@ufu.br (M.F.G.-V.); ghabrielhonorio@ufu.br (G.H.-S.); mario.martins@ufu.br (M.M.M.); lrgoulart@ufu.br (L.R.G.); 2 Department of Biological Sciences, Laboratory of Bioinformatics and Computational Chemistry, State University of Southwest of Bahia (UESB), Jequié 45205-490, Brazil; bandrade@uesb.edu.br; 3 Post-Graduation Program in Genetics and Biochemistry, Laboratory of Nanobiotechnology—Dr Luiz Ricardo Goulart, Federal University of Uberlândia (UFU), Uberlândia 38408-100, Brazil; iarasoares@ufu.br (I.P.S.); ana.notario@ufu.br (A.F.O.N.); 4 Institute of Chemistry, Federal University of Uberlândia (UFU), Uberlândia 38408-100, Brazil; munoz@ufu.br; 5 Institute of Biosciences, Languages, and Exact Sciences (Ibilce), São Paulo State University (Unesp), São José do Rio Preto 15054-000, Brazil; jardim@ufu.br; 6 Laboratory of Antiviral Research, Department of Microbiology, Institute of Biomedical Sciences, Federal University of Uberlandia (UFU), Uberlândia 38408-100, Brazil; 7 Department of Pulmonology, School of Medicine, Federal University of Uberlandia (UFU), Uberlândia 38408-100, Brazil; thulio.cunha@ufu.br; 8 Faculty of Computing, Federal University of Uberlandia (UFU), Uberlândia 38408-100, Brazil; mgcarneiro@ufu.br

**Keywords:** biosensors, COVID-19, bio-inspired peptides, artificial intelligence, salivary diagnostics, electrochemical detection

## Abstract

Developing affordable, rapid, and accurate biosensors is essential for SARS-CoV-2 surveillance and early detection. We created a bio-inspired peptide, using the SAGAPEP AI platform, for COVID-19 salivary diagnostics via a portable electrochemical device coupled to Machine Learning algorithms. SAGAPEP enabled molecular docking simulations against the SARS-CoV-2 Spike protein’s RBD, leading to the synthesis of Bio-Inspired Artificial Intelligence Peptide 1 (BIAI1). Molecular docking was used to confirm interactions between BIAI1 and SARS-CoV-2, and BIAI1 was functionalized on rhodamine-modified electrodes. Cyclic voltammetry (CV) using a [Fe(CN)_6_]^3−/4^ solution detected virus levels in saliva samples with and without SARS-CoV-2. Support vector machine (SVM)-based machine learning analyzed electrochemical data, enhancing sensitivity and specificity. Molecular docking revealed stable hydrogen bonds and electrostatic interactions with RBD, showing an average affinity of −250 kcal/mol. Our biosensor achieved 100% sensitivity, 80% specificity, and 90% accuracy for 1.8 × 10⁴ focus-forming units in infected saliva. Validation with COVID-19-positive and -negative samples using a neural network showed 90% sensitivity, specificity, and accuracy. This BIAI1-based electrochemical biosensor, integrated with machine learning, demonstrates a promising non-invasive, portable solution for COVID-19 screening and detection in saliva.

## 1. Introduction

SARS-CoV-2 is the virus that causes COVID-19, a disease that has resulted in over 775 million cases worldwide and about 7 million deaths [1]. The disease conditions range from asymptomatic to severe respiratory symptoms [2]. Aside from COVID-19 symptoms, it is possible to identify the long-term repercussions of SARS-CoV-2 virus infection in multiple organ systems, such as shortness of breath, cough, chest and joint pains, palpitations, myalgia, smell and taste dysfunctions, headache, cognitive and mental impairments, and gastrointestinal and cardiac issues [3]. Testing on nasopharyngeal swabs or saliva is commonly used for COVID-19 detection, with saliva representing a non-invasive alternative [4]. Although qRT-PCR offers adequate sensitivity and specificity, it has limited bedside application and is time-consuming [4,5]. Furthermore, the early detection of COVID-19 is critical for reducing its spread and mortality rates [6]. To address these issues, point-of-care devices have been developed for rapid diagnosis [5]. However, resource shortages and the need for specific, sensitive, affordable, and non-invasive portable testing methods persist [7].

In this context, electrochemical biosensors provide various advantages for salivary diagnostics, including shorter reaction times, simplicity of use, enhanced affinity, and increased sensitivity [8]. These biosensors have successfully detected low levels of salivary biomarkers, providing a simple and rapid diagnostic procedure [9]. Regarding the COVID-19 pandemic, electrochemical biosensors have shown promise to detect the disease in saliva [10]. Some electrochemical biosensors have been created for the sensitive detection of SARS-CoV-2 at multiple stages of infection [11,12]. This detection is generally performed by detection based on antibody functionalization in electrochemical electrodes to detect viral proteins in saliva [10,11,12,13]. However, chemically synthesized short peptides offer competitive cost advantages and are easier to produce using recombinant bacteria compared to antibodies, which can improve the democratization of salivary diagnostics in low-income and middle-income countries with more non-invasive options in the benchmark diagnostic availability.

Considering that viral transmission can occur through saliva and SARS-CoV-2 is composed of three structural proteins—Spike (S), matrix (M), and envelope (E) [14]—the detection of these proteins in non-processed saliva may represent an even faster diagnostic tool. From this perspective, the receptor binding domain (RBD) in the S protein interacts with the host cellular receptor to permit viral entrance [15]. Thus, detecting the S-RBD motif represents a target on which an especially tailored modified electrochemical sensor can distinguish the virus in saliva and allow COVID-19 detection. Herein, selecting peptides that bind to S-RBD presents a promising field in developing accurate detection tools for SARS-CoV-2 [16]. It also poses a significant challenge considering the rise of new variants—Omicron, for instance, presents several structural alterations, 15 of which are focused on the RBD [17]. From this point of view, artificial-intelligence-based tools and applications have also been proposed to improve accuracy [18].

Evaluating the molecular docking of several peptides with a specific target is time-consuming and costly, so performing numerical simulations for the optimal solution is unaffordable. Therefore, the supervised machine learning (ML) algorithm based on the Surrogate-assisted optimization model is an attractive tool for these problems [19]. Supervised ML is an effective tool for peptide discovery and design, providing faster and more efficient predictions of therapeutic peptide value [20]. Supervised ML has been used in antimicrobial peptides to quickly screen sequence space and direct tests to promising candidates with high putative activity [21]. Moreover, peptides have been designed to bind to SARS-CoV-2 viral proteins by mimicking human angiotensin-converting enzyme 2 (hACE2) receptors using new computational methods [22]. Supervised ML has also been employed to anticipate de novo sequences for viral infectivity improvement, resulting in the identification of short functional self-assembling peptides [23]. Computational techniques have been created for identifying and optimizing possible peptide hits from viral envelope proteins, with a particular emphasis on preventing the fusion process, as well [24] as demonstrating the potential of machine learning in peptide creation for antiviral medicine.

Considering that the early detection of SARS-CoV-2 is imperative for controlling the spread of COVID-19 and traditional diagnostic methods, while effective, are often costly and time-consuming, there is a pressing need for portable, non-invasive, low-cost, and rapid diagnostic tools that can be deployed widely worldwide. Our study aimed to develop an electrochemical biosensor that utilized bio-inspired peptides selected and redesigned using AI coupled to machine learning algorithms to detect SARS-CoV-2 in saliva.

## 2. Materials and Methods

### 2.1. Peptide Development

The Surrogate-Assisted Genetic Algorithm for Peptide Evaluation and Prediction (SAGAPEP) framework [25] was used in a systematic, multi-stage approach to evaluate and find peptides with high binding potential. The approach began with collecting a dataset including 296 peptides; each represented as a linear sequence of amino acids and their relative interaction energies with the SARS-CoV-2 Spike protein [26].

### 2.2. Molecular Docking

Docking calculations were performed by HPEPDOCK 2.0 [27] using the SARS-CoV-2 Spike and the proposed peptide, restricted to the receptor binding domain region (RBD). The affinity energies were identified according to the peptide positioning inside the expected binding site, with the more negative values being the best ones. Then, a 3D interaction map was generated by the Protein–Ligand Interaction Profiler server (PLIP) [28], for checking what bond types were being formed between these molecules. In addition, we generated complex and 3D map figures using Pymol 3.1 [29].

### 2.3. Experimental Procedures

Cyclic voltammograms were recorded using KCl (Sigma-Aldrich, Saint Louis, MO, USA) solution (0.5 mol L^−1^) as supporting electrolyte and 5.0 mmol L^−1^ [Fe(CN)_6_]^3−/4−^ (Sigma-Aldrich, Saint Louis, MO, USA) redox probe as an analysis solution [30]. In the described cyclic voltammetry (CV) experiment, the equilibration time (*t equilibration*) was set to 2 s to stabilize the system before measurements began. The initial potential (*E_begin*) was set at −0.3 V, and the scan was initiated from this potential. The voltage was then cycled to the first vertex potential (*E_vertex1*) of −0.3 V, up to the second vertex potential (*E_vertex2*) of 0.3 V, creating a triangular wave. The step potential (*E_step*) was 0.002 V, allowing for satisfactory resolution of the potential changes. The scan rate was 0.075 V/s, determining the speed at which the potential was swept from the initial to the vertex and back, ensuring detailed capture of the electrochemical behavior of the system [31]. The analyses were performed on a portable potentiostat, Palmsens3 (Palmsens, Houten, The Netherlands).

### 2.4. Screen-Printed Electrode Modification

Screen-printed carbon electrodes—SPEs (DropSens DRP-110, Metrohm, Herisau, Switzerland)—were modified in two steps to improve detection capabilities. Rhodamine 6G (R6G) (Sigma-Aldrich, Saint Louis, MO, USA) [32] was initially administered to the electrodes by adding 5 µL of a 0.5 mg/mL solution to the working electrode. It was followed by five CV measurements with a 5:5 mmol L^−1^ [Fe(CN)_6_]^3−/4−^ in 0.5 mol L^−1^ KCl solution and one more CV reading under the same circumstances. The electrodes were then modified by adding 5 µL of Bio-Inspired Artificial Intelligence Peptide 1 (BIAI1) at 1 mg/mL. Five CV measurements were performed identically to assess the electrochemical behavior after modification, followed by one additional CV reading using the Fe(CN)_6_]^3−/4−^ solution. This step-by-step modification was intended to analyze and compare the electrochemical reactions produced by each modification agent [33].

### 2.5. Sample Preparation

In the first round of assays, a pseudotyped vesicular stomatitis virus (VSV) with the glycoprotein gene (G) substituted with the Spike protein of SARS-CoV-2 (VSV-eGFP-SARS-CoV-2-S) was used. This virus was serially diluted (1:2) in Dulbecco’s modified Eagle’s medium (DMEM; Sigma-Aldrich, Saint Louis, USA), resulting in a range of dilutions from 1.44 × 10^5^ ffu to 2.25 × 10^3^ ffu [34].

Later, saliva samples from healthy, asymptomatic patients were obtained and centrifuged at 3000 rpm for 15 min at 4 °C, with the supernatant collected and pooled. VSV-eGFP-SARS-CoV-2-S was combined with pooled saliva to produce ten infected samples with 1.8 × 10^4^ ffu each and ten control samples free of the virus [35].

Finally, saliva was taken from 20 patients with flu-like symptoms, either COVID-19-positive (ten samples) or -negative (ten samples). A quantity of 5 µL of each sample was used for the assays for all phases.

### 2.6. Data Analysis

Repeatability and reproducibility were calculated by measuring the electrochemical signals of the biosensor under identical conditions (repeatability) and under varying conditions such as with different operators and days (reproducibility). The coefficients of variation were determined from the mean and standard deviation of the measurements. The limit of detection (LOD) and limit of quantification (LOQ) were estimated using the standard deviation of the blank signal and the slope of the calibration curve (SSS) based on the equations LOD=3.3×σ/S and LOQ=10×σ/S.

The classification was tested with state-of-the-art machine learning algorithms, including Support Vector Machine (SVM), AdaBoost, Random Forest, Neural Network, Gradient Boosting, and Naive Bayes [35]. These algorithms were selected based on their performance during model training. To analyze the predictive performance of these algorithms, ten-times-stratified cross-validation was used. The samples were divided into ten subsets; in each iteration, nine subsets were used to train the algorithm while one subset was exclusively used to test it [35]. This process ensured that each subset was part of the test set once. Additionally, the procedure was repeated three times with different sample configurations to achieve a closer estimate of the actual performance of the models, resulting in a total of thirty executions.

Three performance metrics consolidated in the literature were used to measure the results obtained: sensitivity, specificity, and accuracy. Sensitivity, or the valid positive rate, was the proportion of positive cases (e.g., COVID-19 positive) correctly classified. Specificity, or the valid negative rate, was the proportion of negative cases (e.g., COVID-19 negative) correctly classified. Accuracy was defined as the total number of samples correctly classified, considering both true positives and true negatives [36].

## 3. Results

### 3.1. Molecular Docking Interactions

**Figure 1:**
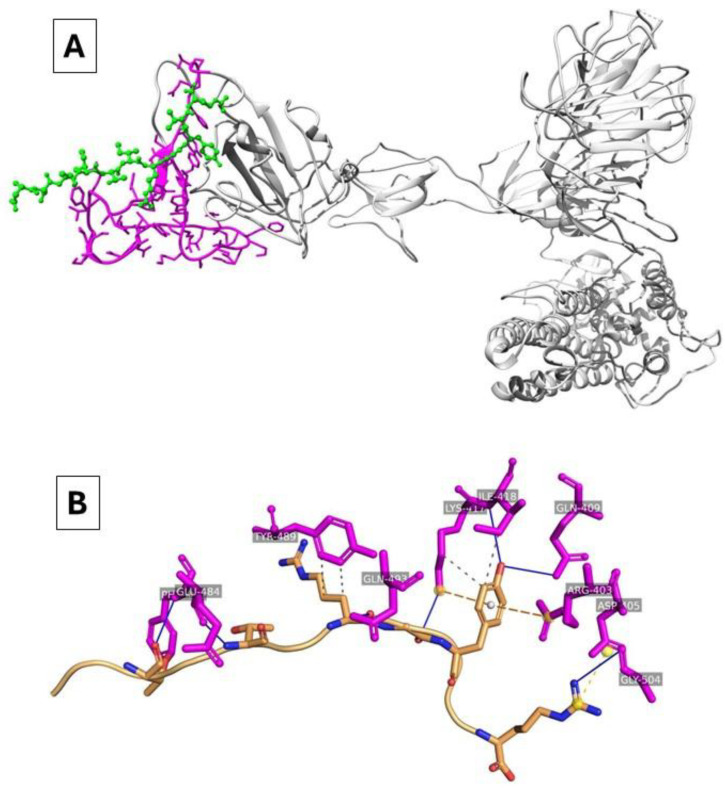
(**A**) Three-dimensional docking representations for BIAI1 interacting with SARS-CoV-2 Spike-RBD. The Spike protein is represented in grey, BIAI1 in green, and RBD in pink. (**B**) Three-dimensional interaction map for BIAI with SARS-CoV-2 Spike-RBD. Hydrogen bonds are presented as dotted lines between the peptides and the Spike-RBD complex.

The peptide formed different hydrogen bonds with the RBD amino acids ASP405, GLN409, LYS417, ILE418, GLU484, PHE486, GLN493, and GLY504, which suggests the good stability of this complex with an average affinity energy of −250 Kcal/Mol. In addition, electrostatic interactions were identified with the residues ASP403, LYS417, ILE418, PHE486, and TYR489 (Figure 1).

### 3.2. Modification of Screen-Printed Electrodes

**Figure 2:**
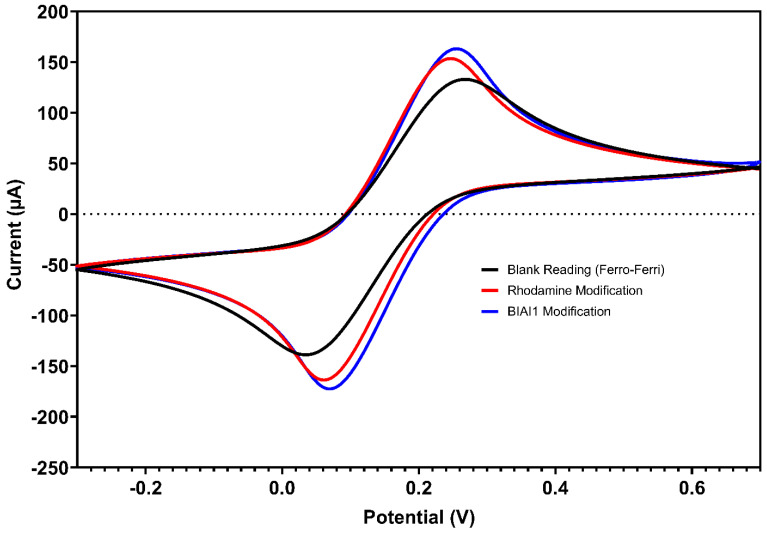
Cyclic voltammograms of a bare screen-printed electrode (black line), electrode modified with R6G at 0.5 mg/mL (red line), and electrode modified with BIAI1 (blue line), using 5.0 mmol L^−1^ [Fe(CN)_6_]^3−/4−^ in KCl 0.5 mol L^−1^. Scans: −0.3 to 0.7 V, E step: 0.002V, and scan rate: 0.075 V/s.

Screen-printed carbon electrodes were successfully modified in two steps to improve detection capabilities as this may have increased the electrode area [37]. R6G was initially used by adding 5 µL of a 0.5 mg/mL solution to the working electrode. This was followed by the modification by adding 5 µL of Bio-Inspired Artificial Intelligence Peptide 1 (BIAI1) at 1 mg/mL (Figure 2).

Figure 2 shows the scan for the [Fe(CN)_6_]^3−/4−^ solution at a SPE any surface modification (blank). The modification with R6G was essential for the construction of the biosensor, acting as an intermediary in the fixation of the peptides. After the application of R6G on the SPE surface, five voltammetric scans in the presence of [Fe(CN)_6_]^3−/4−^ solution were performed. The fifth cyclic voltammogram showed a current increase (red line in Figure 2) in comparison with the blank experiment (absence of R6G). The same process was carried out in the subsequent step for peptide fixation. After peptide incorporation on the SPE surface, a slight current increase was modified, which confirmed the electrode modification. The data for SPE modification can be found in Table 1.

### 3.3. Biosensor Performance

**Figure 3:**
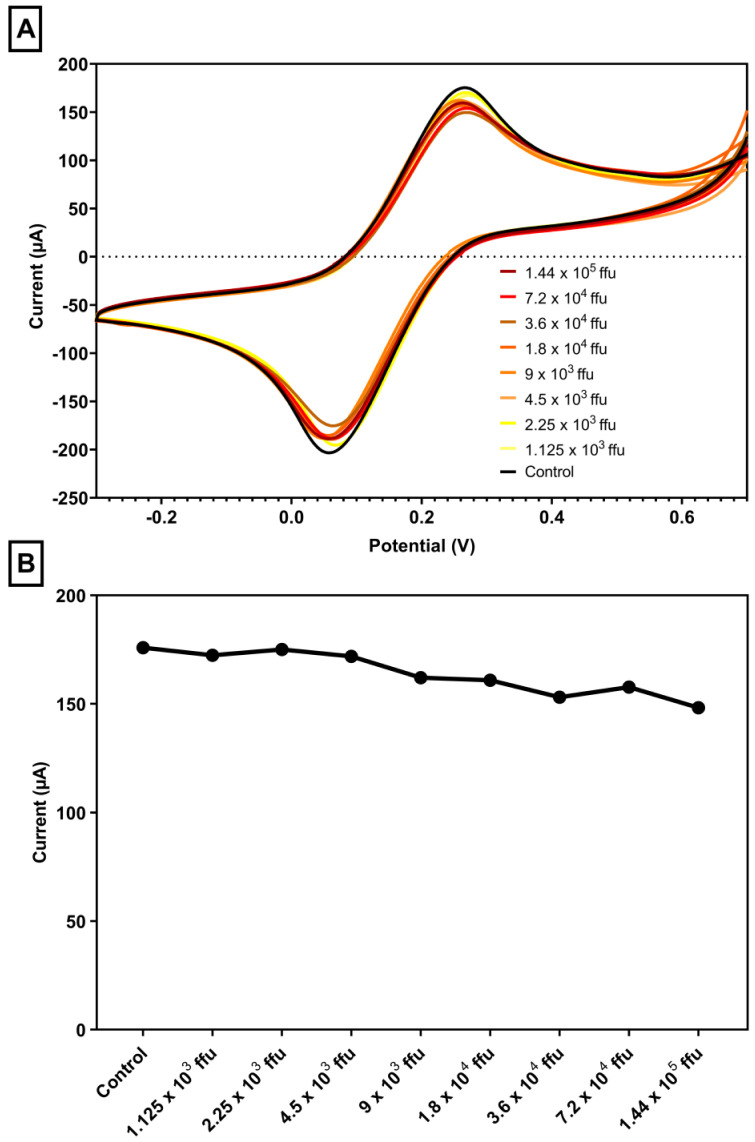
(**A**) Voltammograms of serial dilutions stratified by viral load. Concentration of 1.44 × 10^5^ focus-forming units down to 1.125 × 10^3^. Analysis was performed using 5.0 mmol L^−1^ [Fe(CN)_6_]^3−/4−^ in 0.5 mol L^−1^ KCl. Scans: −0.3 to 0.7 V, E step: 0.002V, and scan rate: 0.075 V/s. (**B**) Peak current values: viral load. Scan rate: 50 mV s^−1^. Analysis was performed using 5.0 mmol L^−1^ [Fe(CN)_6_]^3−/4−^ in 0.5 mol L^−1^ KCl. Scans: −0.3 to 0.7 V, E step: 0.002 V, and scan rate: 0.075 V/s.

In the first phase of the experiment, successive dilutions of VSV-eGFP-SARS-CoV-2 were generated in a 1:2 ratio, beginning with an initial concentration of 1.44 × 10^5^ focus-forming units down to 1.125 × 10^3^. Each dilution was examined using a biosensor modified with the R6G and BIAI1 peptides to detect differences in electrochemical signals. The voltammograms illustrate the electrochemical responses for different concentrations of the virus. Each color-coded curve highlights the distinct peak currents corresponding to the respective viral concentrations, demonstrating the sensitivity of the biosensor to varying levels of VSV-eGFP-SARS-CoV-2. The CV measurements revealed separate peaks for each concentration level, with the highest signal occurring at the initial concentration and a progressive drop in peak current with consecutive dilutions (Figure 3). LOD and LOQ values were calculated and can be found in Table 2.

**Figure 4:**
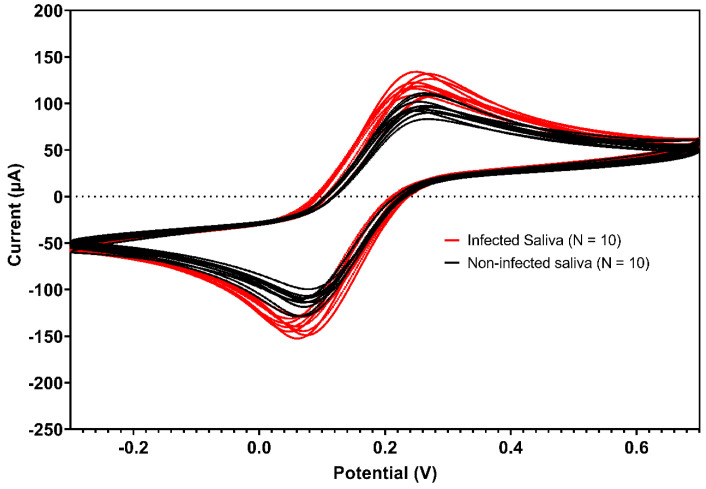
Voltammograms of pooled saliva samples of healthy asymptomatic individuals. The voltammograms illustrate the electrochemical responses for two groups: non-infected saliva, without VSV-eGFP-SARS-CoV-2, in black; infected saliva, with 1.8 × 10^4^ of the virus, in red. Analysis was performed using 5.0 mmol L^−1^ [Fe(CN)_6_]^3−/4−^ in 0.5 mol L^−1^ KCl. Scans: −0.3 to 0.7 V, E step: 0.002 V, and scan rate: 0.075 V/s.

**Figure 5:**
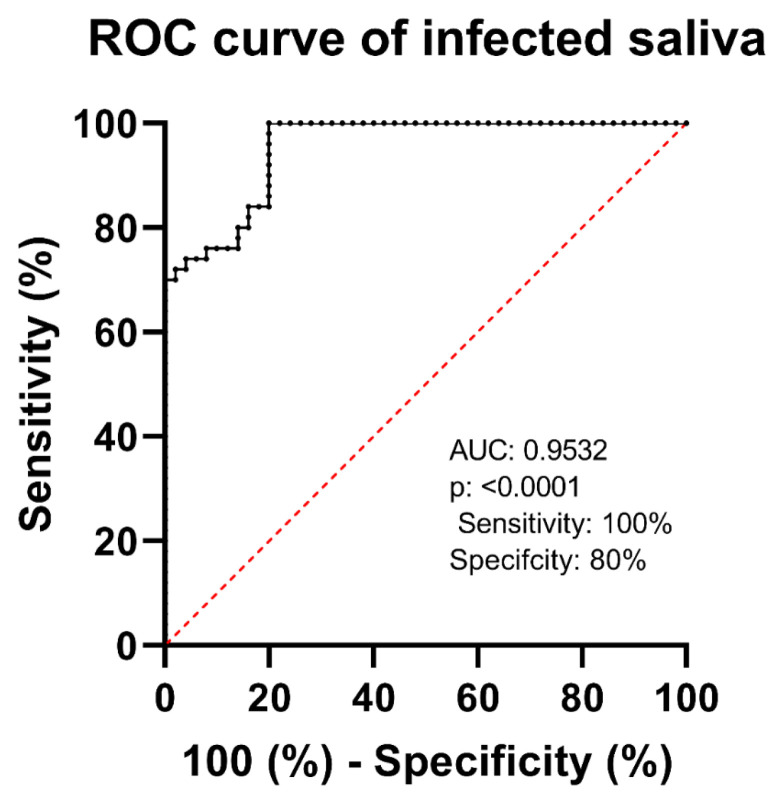
Graph representing the electrochemical sensor diagnostic test effectiveness using ROC curve analysis, producing an AUC of 0.9532 (95% CI: 0.9182 to 0.9882) and a *p*-value < 0.0001.

In the second phase, saliva samples from healthy individuals were collected, pooled, and spiked with the VSV-eGFP-SARS-CoV-2-S virus. Comparison between the voltammograms from the saliva pool, both with and without the virus, demonstrated a clear distinction in peak currents, indicating successful virus detection in saliva (Figure 4). For these samples, the biosensor exhibited a sensitivity of 100% and a specificity of 80%, resulting in an accuracy of 90%. The ROC curve analysis for this phase provided an area under the curve (AUC) of 0.9532, with a standard error of 0.01785 and a *p*-value of <0.0001. These assays demonstrated an attractive efficiency in detecting real positives while minimizing false positives at higher thresholds (Figure 5). The data for the repeatability and reproducibility of the detection of VSV-eGFP-SARS-CoV-2-S can be found in Table 3.

**Figure 6:**
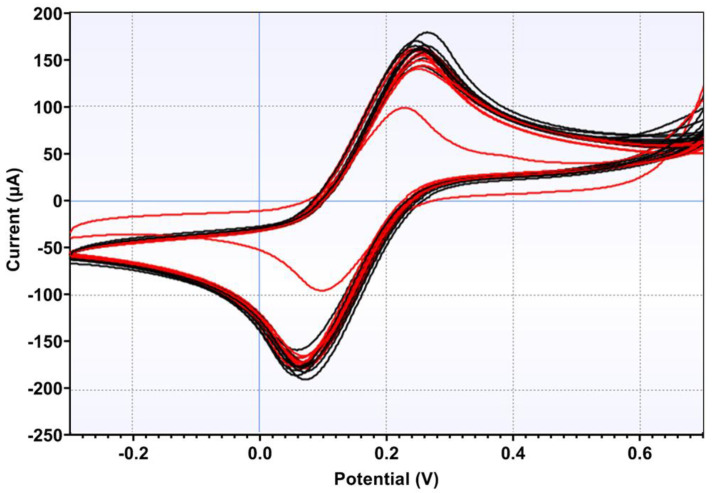
Voltammograms of saliva samples from patients with flu-like symptoms and negative RT-PCR test to detect SARS-CoV-2 (black) and COVID-19 positive patients (red). Analysis was performed using 5.0 mmol L^−1^ [Fe(CN)_6_]^3−/4−^ in KCl 0.5 mol L^−1^. Scans: −0.3 to 0.7 V, E step: 0.002 V, and scan rate: 0.075 V/s.

**Figure 7:**
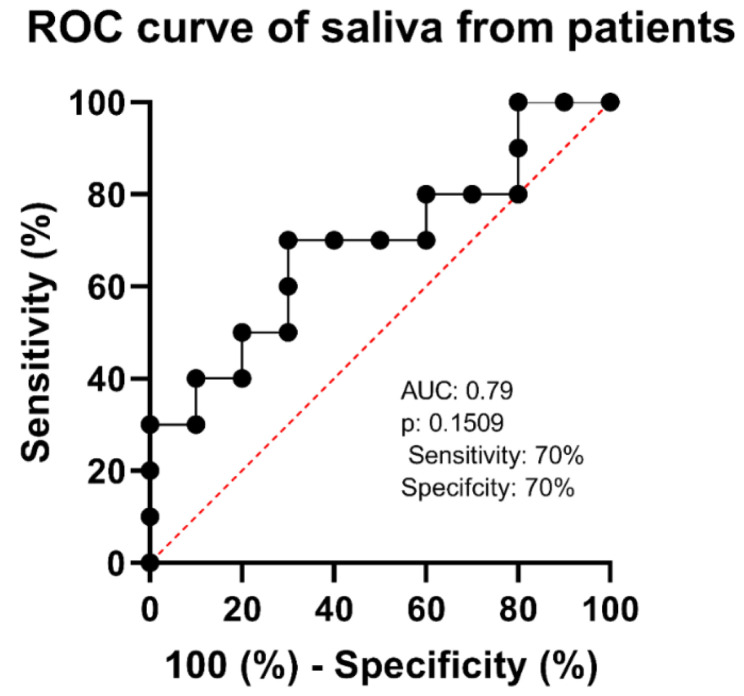
Graph representing the electrochemical sensor diagnostic test effectiveness using ROC curve analysis, producing an AUC of 0.79 with sensitivity and specificity of 70%, a standard error of 0.1213, and a *p*-value of 0.1509.

In the third phase, saliva from patients with flu-like symptoms and negative RT-PCR tests were analyzed to detect COVID-19 and COVID-19 patients. Voltammograms from the negative and positive patients showed a distinction for peak current and potential values between the two groups, suggesting that the virus was successfully detected in the saliva of patients (Figure 6). The ROC curve analysis for oxidation yielded an AUC of 0.79 with a standard error of 0.1213, sensitivity and specificity of 70%, and a p-value of 0.1509, indicating moderate discriminatory power that was not statistically significant (Figure 7). The data for the repeatability and reproducibility of the detection of SARS-CoV-2 in comparison with other symptomatic patients can be found in Table 4.

### 3.4. Discrimination Analysis

Artificial intelligence tools were applied to classify and discriminate salivary voltammogram samples from positive COVID-19-symptomatic patients and negative COVID-19-symptomatic patients more quickly and with better reliability, most notably machine learning algorithms. The classification of salivary voltammogram data was tested using the SVM, AdaBoost, Random Forest, Neural Network, Gradient Boosting, and Naive Bayes algorithms. The results obtained in these analyses indicate that the best discrimination was achieved using a Neural Network (Table 5).

The classification of salivary voltammogram data by the Neural Network algorithm showed a sensitivity of 90%, specificity of 90%, and accuracy of 90% when comparing positive COVID-19-symptomatic patients and negative COVID-19-symptomatic patients. This high level of accuracy demonstrated the potential of using electrochemical biosensors combined with advanced machine learning techniques for the non-invasive diagnosis of COVID-19.

**Figure 8:**
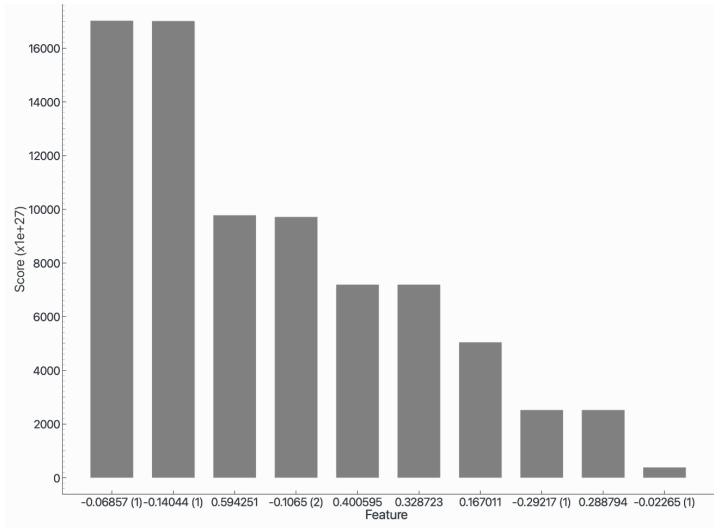
SHAP demonstration used by the Neural Network computational model with the raw data of the reduction curve. The potentials of −0.06857 V, −0.14044 V, 0.594251 V, and −0.1065 V were the main attributes used in the applications of the algorithm. The regions of 0.400595 V, 0.328723 V, 0.167011 V, −0.29217 V, 0.288794 V, and −0.02265 V were also used for this differentiation.

The Shapley Additive Explanations (SHAP) method was applied to the main points of the voltammogram that contributed significantly to the discrimination of the samples, distinguishing them as positive or negative for COVID-19 (Figure 8). In general, the main points of discrimination in the results were present in the peak region of the reduction curve.

## 4. Discussion

We presented one bio-inspired peptide selected and redesigned using AI, explicitly designed to strongly interact with the S-RBD motif of SARS-CoV-2, to be used in an electrochemical biosensor to detect COVID-19 when coupled with machine learning algorithms. Due to their excellent specificity and sensitivity, peptides are increasingly being employed in electrochemical devices to detect viruses. For example, electrochemical-peptide-based sensors for the HIV and West Nile Virus can detect an array of clinically relevant viral concentrations [38,39]. Peptides have also been used for specific viral antigens such as the influenza virus antigen and avian influenza virus [40,41]. Even more complex approaches have been tested, utilizing antibody-coated peptide nanotubes for herpes simplex virus type 2 [42]. These findings show that peptides can improve the effectiveness of electrochemical sensors to detect viruses. Still, our study reported the first recorded application of an electrochemical biosensor technology based on screen-printed carbon electrodes functionalized with an AI-generated synthetic peptide to detect the SARS-CoV-2 Spike protein, presenting a viable approach to quick and inexpensive, point-of-care diagnostics.

The biosensor displayed good sensitivity by detecting different concentrations of VSV-eGFP-SARS-CoV-2 in affinity assays. Serial dilutions were made in a 1:2 ratio, starting from an initial concentration of 1.44 × 10⁵ ffu and decreasing to 2.23 × 10³. The CV measurements indicated separate peaks for each concentration level, with the highest signal detected at the starting concentration. The peak currents were reduced with each consecutive dilution, demonstrating the capability of this biosensor to differentiate between different viral loads. Several electrochemical technologies have been developed to detect viral concentrations, with benefits including label-free detection, quick on-site analysis, and high sensitivity [43,44]. An electrochemical sensor based on gold nanoparticles was created to detect HIV-1 virus-like particles label-free, with a detection range of 600 fg/mL to 375 pg/mL, allowing direct electron flow between the virus and the electrode surface [43].

Similarly, a sponge-based electrochemical sensor that can detect the H1N1 virus with a limit of detection of 0.4 TCID50/mL and a practical concentration range of 1–106 TCID50/mL had been presented before [44]. Regarding SARS-CoV-2 detection, a previous study used a simple electrochemical biosensor device coupled with screen-printed gold electrodes functionalized with a particular synthetic peptide to detect the interaction between the peptide and the SARS-CoV-2 Spike protein using electrochemical impedance spectroscopy [45]. This last biosensor was tested using commercial Spike protein solutions and lysed SARS-CoV-2 particles, in opposition to our study, where we used entire viral particles. In this context, our study represented an advancement in the detection of the virus, considering that interaction with other viral proteins might affect the virus’s binding ability to the sensor [46].

Pooled saliva samples from healthy individuals injected with VSV-eGFP-SARS-CoV-2-S produced unique voltammogram peaks, suggesting successful viral identification in the saliva matrix. The biosensor achieved 100% sensitivity and 80% specificity, yielding 90% accuracy. ROC curve research revealed an AUC of 0.9532, demonstrating the sensor’s excellent efficacy in detecting real positives while limiting false positives, making it a dependable instrument for viral detection in saliva samples. Several investigations have shown that either pooled or artificial saliva may be used as a matrix to identify SARS-CoV-2. This has been investigated using immunochromatographic and automated molecular tests, which have yielded encouraging findings but not offered comprehensive sensitivity, specificity, or accuracy values [47,48]. Studies have also looked at the stability of SARS-CoV-2 RNA in saliva and used an artificially intelligent nanopore for fast testing [49,50]. Overall, our biosensor performance with pooled saliva spiked with VSV-eGFP-SARS-CoV-2-S provided clear and quantifiable results, demonstrating high sensitivity and specificity compared to the literature.

Saliva samples from symptomatic individuals were tested using our AI-generated peptide-based electrochemical sensor. The ROC curve analysis produced an AUC of 0.69, showing moderate discriminating power; however, the *p*-value of 0.1509 that indicates this discrimination was not statistically significant. Sensitivity and specificity were 70%, emphasizing the potential of saliva analysis for COVID-19 identification and the need for additional diagnostic refinement and validation. In this context, the composition of saliva in infected individuals has a considerable influence on the outcomes of salivary diagnostic techniques. Saliva includes a variety of biomolecules, such as DNA, RNA, proteins, and metabolites, which can change in the presence of illness [51]. However, this changed composition of saliva in infected individuals might impair the accuracy of salivary diagnostic techniques, potentially resulting in false-positive or false-negative findings [51].

When compared to traditional antibody-based electrochemical sensors for detecting SARS-CoV-2 infections, our bio-inspired peptide platform can represent a significant advancement in the analysis of clinical samples. Antibody-based systems frequently detect host immune responses rather than the virus itself, which delays diagnosis, cannot confirm active infections, and fails to provide quantitative information on viral load or contagiousness, limiting these systems’ effectiveness for early and precise detection [52]. Furthermore, modifying electrodes with antibodies is generally more expensive than using peptides, primarily due to the greater complexity and cost associated with synthesizing larger biomolecules. Antibody production typically relies on advanced cell culture systems and rigorous purification protocols whereas peptides often can be synthesized more efficiently and cost-effectively through established chemical methods [53]. Additionally, while antibody-based sensors have been well established in the literature, their sensitivity and reproducibility can be particularly challenging when detecting active viral infections owing to inherent trade-offs between the binding efficiency of capture agents and the accuracy of the electrochemical measurement [54]. Moreover, the peptide-based design improves the electrochemical stability of the biosensor, addressing the degradation and fouling issues frequently encountered with antibody-functionalized electrodes. This advancement ensures more reliable electrochemical responses under varied conditions and highlights the potential of peptide-based platforms for scalable and high-performance diagnostic applications [55]. In this context, our biosensor based on bio-inspired peptides, while demonstrating comparable sensitivity and specificity to existing systems, introduces a novel and cost-effective approach to biosensor development.

Electrochemical sensors have attractive diagnostic applications but are limited by electrode fouling, a low signal-to-noise ratio, and chemical interference [56]. These challenges may be addressed using ML, which improves sensitivity, repeatability, and accuracy in data analysis. ML simultaneously measures several toxicants [57] and enhances cancer biomarker identification [58]. The detection of ischemic heart disease was demonstrated over ten years ago, which attained performance levels equivalent to doctors while significantly boosting sensitivity and specificity [59,60]. Deep learning and machine learning algorithms have also been used for illness prediction and medical diagnostics [61,62]. However, ML applications need big datasets and meticulous picture capture [63]. In our study, we used artificial intelligence algorithms to analyze the spectra of the positive and negative samples, increasing sensitivity, specificity, and accuracy to 90%. Previous research had highlighted the potential of these algorithms in analyzing multi-component and Raman spectra [64,65].

The ability of electrochemical biosensors to detect various viral diseases has improved due to recent developments in functional nanomaterials [66]. In this context, the limit of detection and selectivity for harmful microorganisms and nucleic acids have been enhanced by signal amplification techniques [67]. With their benefits in speed, sensitivity, and affordability, electrochemical biosensors have demonstrated promise for point-of-care testing [68,69]. For future pandemic preparedness, emerging-technologies-based biosensors present interesting options [70].

Further emphasis has been placed on using algorithms in analyzing electromagnetic emission spectrum data [71]. Nevertheless, the application of machine learning is still embryonic, and our approach represents a novel mechanism for analyzing the spectra generated by CV. Algorithms have demonstrated tremendous promise in assessing electrode reaction processes and interpreting data from polarography, linear scanning voltammetry, and electrochemical impedance spectroscopy [72,73]. Machine learning has also been used to model smartphone-based electrochemiluminescence sensor data and pinpoint accurate signs of localized corrosion [74,75]. Despite advances, the commercialization of point-of-care devices remains challenging due to the requirement for expensive reagents, equipment, and skilled staff [69]. Although further studies with a higher number of samples in multicentric cohorts are pivotal for additional validation, this present study emphasized the importance of AI in improving electrochemical analysis, and our research contributes to further its application to a different branch of analytic electrochemistry for salivary diagnostics.

In summary, our study highlighted several advantages of AI-generated peptides over traditional antibody-based sensors, including reduced production costs, enhanced stability, and adaptability to emerging targets, as previously reported in the literature [76,77]. While antibody-based sensors often require complex and expensive production pipelines, our peptide-based system leverages computational design for rapid development [78]. Additionally, the integration of machine learning algorithms that we used enables precise signal interpretation, further distinguishing our platform, as has been previously reported [79,80].

## 5. Conclusions

The functionalization of the electrode with BIAI1, a bio-inspired peptide selected and redesigned using AI, to create an electrochemical biosensor supported by the Neural Network machine learning algorithm offers a groundbreaking tool for salivary diagnostics in a post-pandemic environment. This portable, non-invasive, and scalable technology not only facilitates COVID-19 detection but also underscores the growing importance of saliva as a diagnostic medium for the rapid screening and early detection of SARS-CoV-2 and other infectious diseases, contributing to improved public health surveillance and preparedness.

## Figures and Tables

**Table 1:** Mean, standard deviation, repeatability, and reproducibility values of the electrochemical procedures for SPE modifications.

	Mean	Standard Deviation	Repeatability *	Reproducibility *
**Blank Reading**	46.98979	3.224133	6.861348	4.385376
**R6G Modification**	46.71211	4.19041	8.970715	4.979766
**BIAI1 Modification**	52.85969	3.613036	6.835144	4.866002

* Repeatability and reproducibility are represented as their coefficients of variation in percentage.

**Table 2:** Limit of Detection (LOD) and Limit of Quantification (LOQ) from the de serial dilutions performed on the first phase of the experiment.

**LOD**	1.61 × 10^4^ ffu
**LOQ**	4.89 × 10^4^ ffu

**Table 3:** Mean, standard deviation, repeatability, and reproducibility values of the electrochemical procedures for viral detection in spiked saliva.

	Mean	Standard Deviation	Repeatability *	Reproducibility *
**Controls**	97.10048056	8.140980015	8.384077987	2.209871449
**Infected saliva**	119.443717	8.893037898	7.445379397	2.201690182

* Repeatability and reproducibility are represented as their coefficients of variation in percentage.

**Table 4:** Mean, standard deviation, repeatability, and reproducibility values of the electrochemical procedures for viral detection in saliva comparing positive and negative patients.

	Mean	Standard Deviation	Repeatability *	Reproducibility *
**Negative Patientes**	160.5768	9.907850286	6.170162991	12.08960789
**Positive Patients**	150.31038	12.05649675	8.021067309	15.10250925

* Repeatability and reproducibility are represented as their coefficients of variation in percentage.

**Table 5:** Machine learning algorithms applied in salivary voltammograms to discriminate positive COVID-19-symptomatic patients from negative COVID-19-symptomatic patients.

Data Used	Algorithm	Accuracy	Sensitivity	Specificity
Raw Data (Oxidation and Reduction Curves)	SVM	0.75	0.6	0.9
	Random Forest	0.75	0.7	0.8
	AdaBoost	0.7	0.6	0.8
	Neural Network	0.75	0.8	0.7
	Gradient Boosting	0.7	0.7	0.7
	Naive Bayes	0.75	0.8	0.7
Raw Data (Oxidation Curve)	SVM	0.5	0.5	0.5
	AdaBoost	0.65	0.7	0.6
	Random Forest	0.5	0.5	0.5
	Neural Network	0.5	0.6	0.4
	Gradient Boosting	0.55	0.6	0.5
	Naive Bayes	0.35	0.4	0.3
Raw Data (Reduction Curve)	SVM	0.6	0.2	1.0
	AdaBoost	0.7	0.7	0.7
	Random Forest	0.75	0.7	0.8
	Neural Network	0.9 *	0.9 *	0.9 *
	Gradient Boosting	0.7	0.7	0.7
	Naive Bayes	0.75	0.8	0.7

* Highest values for accuracy, sensitivity and specificity.

## Data Availability

Data are available upon request.
